# Inter-rater reliability of AMSTAR is dependent on the pair of reviewers

**DOI:** 10.1186/s12874-017-0380-y

**Published:** 2017-07-11

**Authors:** Dawid Pieper, Anja Jacobs, Beate Weikert, Alba Fishta, Uta Wegewitz

**Affiliations:** 1 Institute for Research in Operative Medicine, Witten/Herdecke University, Ostmerheimer Str. 200 (Building 38), 51109 Cologne, Germany; 2 The Federal Joint Committee (G-BA), Wegelystr. 8, 10623 Berlin, Germany; 3 Federal Institute for Occupational Safety and Health (BAuA), Nöldnerstr. 40-42, 10317 Berlin, Germany

**Keywords:** Systematic review, AMSTAR, Reliability and validity, Observer variation, Clinimetrics, Measurement properties

## Abstract

**Background:**

Inter-rater reliability (IRR) is mainly assessed based on only two reviewers of unknown expertise. The aim of this paper is to examine differences in the IRR of the *Assessment of Multiple Systematic Reviews* (AMSTAR) and R(evised)-AMSTAR depending on the pair of reviewers.

**Methods:**

Five reviewers independently applied AMSTAR and R-AMSTAR to 16 systematic reviews (eight Cochrane reviews and eight non-Cochrane reviews) from the field of occupational health. Responses were dichotomized and reliability measures were calculated by applying Holsti’s method (r) and Cohen’s kappa (κ) to all potential pairs of reviewers. Given that five reviewers participated in the study, there were ten possible pairs of reviewers.

**Results:**

Inter-rater reliability varied for AMSTAR between *r* = 0.82 and *r* = 0.98 (median *r* = 0.88) using Holsti’s method and κ = 0.41 and κ = 0.69 (median κ = 0.52) using Cohen’s kappa and for R-AMSTAR between *r* = 0.77 and *r* = 0.89 (median *r* = 0.82) and κ = 0.32 and κ = 0.67 (median κ = 0.45) depending on the pair of reviewers. The same pair of reviewers yielded the highest IRR for both instruments. Pairwise Cohen’s kappa reliability measures showed a moderate correlation between AMSTAR and R-AMSTAR (Spearman’s ρ =0.50). The mean inter-rater reliability for AMSTAR was highest for item 1 (κ = 1.00) and item 5 (κ = 0.78), while lowest values were found for items 3, 8, 9 and 11, which showed only fair agreement.

**Conclusions:**

Inter-rater reliability varies widely depending on the pair of reviewers. There may be some shortcomings associated with conducting reliability studies with only two reviewers. Further studies should include additional reviewers and should probably also take account of their level of expertise.

**Electronic supplementary material:**

The online version of this article (doi:10.1186/s12874-017-0380-y) contains supplementary material, which is available to authorized users.

## Background

In general terms, measurement can be described as the process of systematically assigning numbers or labels to objects and their properties. In medicine, for example, measurement questions can range from symptoms, physical examinations, laboratory tests and imaging to self-report questionnaires. The measurements obtained can be used as a basis for subsequent decisions (e.g. regarding treatments). It is therefore important that measurements are reliable and valid, aspects which are also referred to as measurement properties. Otherwise, there is a serious risk of imprecise or biased results that could lead to incorrect decisions or conclusions. According to the COnsensus-based Standards for the selection of health Measurement INstruments (COSMIN) initiative, reliability is defined as “the degree to which the measurement is free from measurement error”, and validity is defined as “the degree to which an instrument truly measures the construct(s) it purports to measure” [1].

In the context of evidence-based health care, critical appraisal with respect to risk of bias or methodological quality plays an important role in the carrying out of systematic reviews (SRs), which form the cornerstone of evidence-based medicine. The assessment of risk of bias or methodological quality also constitutes a form of measurement. Depending on the included study types, there are a variety of existing instruments (i.e. measurement tools), such as the Cochrane Risk of Bias Tool for randomized controlled trials [2], QUADAS-2 for diagnostic accuracy studies [3], or the Newcastle-Ottawa Scale for cohort and case-control studies [4], for example. There are also organizations such as the Centre for Evidence-based Medicine (CEBM) in Oxford [5] or the Critical Appraisal Skills Programme (CASP) [6] that offer a whole set of quality measurement tools for various the study designs. For SRs, the *Assessment of Multiple Systematic Reviews* (AMSTAR) tool has become the most widely used tool for assessing methodological quality. It was developed based on the Overview Quality Assessment Questionnaire (OQAQ) [7] and the checklist created by Sacks [8] and consists of 11 items. Another research group revised AMSTAR (R-AMSTAR) in order to quantify methodological quality by assigning a quality score to each SR [9].

A recent systematic review found AMSTAR, but not R-AMSTAR, to have good measurement properties, including inter-rater reliability [10]. However, the authors pointed out that inter-rater reliability was mainly assessed based on only two reviewers and without any information regarding the reviewers’ level of expertise in the included studies. Low inter-rater reliability poses potential problems for users of evidence synthesis products, as users may need to consider whether a review would have reached a different conclusion had the methodological quality been assessed by different assessors [11]. It is important to bear this in mind, as measurement theory states that there can be no validity without reliability. Both factors – number of reviewers and level of expertise – may have an influence on inter-rater reliability and may pose a risk to the validity of SRs, as they could lead to a biased conclusion based on flawed ratings. This aspect has not been investigated in prior studies in the field of evidence-based health care. The aim of this paper is therefore to examine differences in inter-rater reliability between AMSTAR and R-AMSTAR depending on the pair of reviewers.

## Methods

This manuscript is part of a larger project conducted by the author group to investigate differences between AMSTAR [12], R(evised)-AMSTAR [13] and *Preferred Reporting Items for Systematic Reviews and Meta-Analyses* (PRISMA) [14] in SRs in the field of occupational health. There was no a priori developed protocol for this study.

### Study selection

SRs in the field of occupational health were identified via a systematic search in MEDLINE (via PubMed) and the Cochrane Database of Systematic Reviews (via the Cochrane Library) at the end of December 2014. The search strategy for MEDLINE can be found in Additional file 1: Appendix 1. In the Cochrane Database of Systematic Reviews, we screened all SRs belonging to the *Health & safety at work* topic (obtained via the browse by topic function). We included SRs published from 2010 to 2014 which included at least one randomized controlled trial,. As the work forms part of a larger project, the decision was made to include Cochrane Reviews (CRs) and non-Cochrane reviews (nCRs) at a 1:1 ratio. The target sample size was set at 16 based on the availability of resources and timelines. A total of 18 SRs were identified: nine Cochrane reviews and nine non-Cochrane reviews were selected.. SRs were randomly ordered using a computer-generated list, were screened consecutively for relevance, and the first 18 SRs matching the inclusion criteria were selected. From these, the first CR and nCR on the list were selected for a calibration exercise. Both SRs were assessed and ratings were discussed between all of the reviewers in a telephone conference to reach consensus. The results of the telephone conference were collected on an instrument and item basis (i.e. amendments were made for the scoring guidance of AMSTAR and R-AMSTAR, if necessary) and were made available to all reviewers once all reviewers agreed on all amendments.

### Quality assessment

AMSTAR and R-AMSTAR were applied by five reviewers to all 16 SRs in an a priori determined order. The reviewers used the version of AMSTAR available at www.amstar.ca [15] (see Table 1). This version includes scoring guidance in the form of notes on each item. It was ensured that all reviewers used the same version of AMSTAR. Each item was rated by applying the standardized set of four possible responses: “yes”, “no”, “can’t answer” or “not applicable”. With respect to R-AMSTAR, the reviewers used the version provided in the source publication by Kung et al. [9]. This publication fails to provide possible categories of answer, so we opted for the responses “yes”, “no” and “not applicable”. In total, R-AMSTAR consists of 41 items with a maximum quality score of 44. We referred to review protocols where available but did not contact review authors. Once the SRs had been critically appraised by each reviewer, regular telephone conferences were held to reach consensus among all of the reviewers with regard to a final assessment. Consensus conferences were held after all reviewers have completed their assessment of all SRs.

**Table 1:** AMSTAR checklist

1. Was an ‘a priori’ design provided?
2. Was there duplicate study selection and data extraction?
3. Was a comprehensive literature search performed?
4. Was the status of publication (i.e. grey literature) used as an inclusion criterion?
5. Was a list of studies (included and excluded) provided?
6. Were the characteristics of the included studies provided?
7. Was the scientific quality of the included studies assessed and documented?
8. Was the scientific quality of the included studies used appropriately in formulating conclusions?
9. Were the methods used to combine the findings of studies appropriate?
10. Was the likelihood of publication bias assessed?
11. Was the conflict of interest included?

All reviewers had several years of experience in the field of evidence-based health care. Before the study, a short questionnaire was sent to all reviewers so that they could provide a self-assessment of their experience, including questions regarding their work experience (in years), the number of SRs assessed with either AMSTAR, R-AMSTAR or OQAQ, and the number of SRs assessed with any other instruments (e.g. the SIGN checklist). The results of this are documented in Table 2. Furthermore, three reviewers were from the same institution and had worked together on several occasions. One of the three reviewers is leading her working group as a research scientist. The fourth reviewer had worked closely with her on former projects. However, none of the former collaborations related to the critical appraisal of SRs. The last reviewer (research scientist leading his own research group) had no former relationship with any other reviewer. The periods of collaboration between individual reviewers are summarized in Table 3.

**Table 2:** Experience of reviewers

	Reviewer no.				
	1	2	3	4	5
Working experience (in years)	13	7	5	7	7
Number of SRs assessed with either AMSTAR, R-AMSTAR or OQAQ	15	1	100	10	10
Number of SRs assessed with any other tool	35	5	10	5	50

**Table 3:** years of collaboration for each pair

Pair of reviewers	Collaboration (in years)
1&2	0
1&3	0
1&4	5
1&5	3
2&3	0
2&4	3
2&5	0
3&4	0
3&5	0
4&5	3

### Data analysis

For AMSTAR, the responses were dichotomized (“yes” vs. any other score) in order to ensure a high level of comparability with prior studies investigating inter-rater reliability, most of which also dichotomized the responses [10]. Three response categories – “yes”, “no”, and “not applicable” – were available with respect to R-AMSTAR, and these responses were also dichotomized (“yes” scores vs. “no”/“not applicable”) to allow the results of AMSTAR to be compared with those of R-AMSTAR. ‘Yes’ answers always refered to a favourable score.

Given that five reviewers participated in the study, there were ten possible pairs of reviewers (1&2, 1&3, 1&4, 1&5, 2&3, 2&4, 2&5, 3&4, 3&5 and 4&5).

Overall, two reliability measures were calculated. Firstly, we applied the Holsti method (r) [16], which in this case yields a value equal to the raw agreement (i.e. counting the number of times agreement has occurred expressed as percentage). Secondly, we calculated Cohen’s kappa (κ) [17], as this is the most prevalent reliability measure applied in this context. Inter-rater reliability measures were calculated as a mean of all AMSTAR items (based on the value of each item) for the Holsti method (r) and Cohen’s kappa (κ) using the method for nominal scaled data. The same procedure was applied with respect to R-AMSTAR for the Holsti method (r) and Cohen’s kappa (κ), including all 41 items.

A value of *r* > 0.9 represents good agreement under the Holsti method [18]. For the interpretation of Cohen’s kappa, the degree of agreement was categorized as poor (κ < 0), slight (κ = 0.00–0.20), fair (κ = 0.21–0.40), moderate (κ = 0.41–0.60), substantial (κ = 0.61–0.80) or almost perfect (κ = 0.81–1.00) based on generally accepted approaches [19].

Spearman’s rho was calculated as a measure of correlation between AMSTAR and R-AMSTAR scores (two-tailed). Differences between CRs and nCRs were analysed applying the Wilcoxon-Mann-Whitney-Test. All tests were run with a significance level of 0.05.

### Software

Microsoft Excel was used to calculate the reliability measures κ (Cohen’s kappa) and r (Holsti’s method) using a freely available Excel macro for reliability coefficients [20]. All other analyses were conducted using SPSS 21.

## Results

Characteristics of the included reviews (all interventional) can be found in Additional file 2: Appendix 2. The number of included RCTs ranged from 3 to 57 with a median of 9. Meta-analysis was conducted in 10 (6 CRs vs. 4 nCRs) out of 16 SRs. The median number of “yes” items (counting “not applicable” items also as “yes”) was 8.5 (range 4–10) for AMSTAR and 36.5 (range 29–42) for R-AMSTAR. For both instruments, the statistically significant result was that CRs obtained more “yes” items than nCRs (AMSTAR: 9 vs. 5.5, *p* < 0.001; R-AMSTAR: 39 vs. 32.5, *p* < 0.001).

The results for inter-rater reliability per pair of reviewers are presented in Table 4. For AMSTAR, the median inter-rater reliability for the pair of reviewers was 0.52 for κ (range 0.41–0.69), and 0.88 for r (range 0.82–0.98). For R-AMSTAR, the corresponding values were 0.45 for κ (range 0.32–0.67) and 0.82 for r (range 0.77–0.89). Spearman’s rho was calculated as 0.50 (*p* = 0.14) and 0.57 (*p* = 0.08) for κ and r respectively as a measure of the correlation between pairwise inter-rater reliability measures for AMSTAR and R-AMSTAR.

**Table 4:** Pairwise inter-rater reliability of AMSTAR and R-AMSTAR

	AMSTAR		R-AMSTAR	
Pair of reviewers	Cohens κ	Holsti’s r	Cohens κ	Holsti’s r
1&2	0.55	0.87	0.37	0.81
1&3	0.56	0.82	0.45	0.84
1&4	0.47	0.86	0.46	0.84
1&5	0.41	0.82	0.32	0.77
2&3	0.52	0.89	0.49	0.83
2&4	0.69	0.98	0.67	0.89
2&5	0.50	0.88	0.39	0.82
3&4	0.53	0.89	0.67	0.87
3&5	0.43	0.83	0.44	0.80
4&5	0.52	0.88	0.45	0.81
min	0.41	0.82	0.32	0.77
max	0.69	0.98	0.67	0.89
mean	0.52	0.87	0.47	0.83
median	0.52	0.88	0.45	0.82

The mean inter-rater reliability for AMSTAR was highest for item 1 *was an ‘*a priori*’ design provided?* (κ = 1.00) and item 5 *was a list of studies (included and excluded) provided?* (κ = 0.78), with the respective values indicating almost perfect or substantial agreement for AMSTAR. The lowest values were found for item 3 *was a comprehensive literature search performed?*, item 8 *was the scientific quality of the included studies used appropriately in formulating conclusions?*, item 9 *were the methods used to combine the findings of studies appropriate?* and item 11 *was the conflict of interest included?*, which showed only fair agreement (Table 5). Inter-rater reliability could not be calculated for item 6 *were the characteristics of the included studies provided?* and item 7 *was the scientific quality of the included studies assessed and documented?* because of empty cells due to small variations in the ratings (e.g. one rater assesses all 16 reviews with “yes”) except for one pair. Some items revealed a very wide range of inter-rater reliability values depending on the pair of reviewers. This is especially true for items 3 and 8, where the difference between the minimum and maximum is greater than 0.8. Interestingly, three pairs of reviewers managed to agree fully on item 8 (i.e. reliability = 1.00).

**Table 5:** Pairwise inter-rater reliability (Cohen’s kappa) for AMSTAR on item-level

Pair	1&2	1&3	1&4	1&5	2&3	2&4	2&5	3&4	3&5	4&5	Mean	Median
I1: Was an ‘a priori’ design provided?	**1.00**	**1.00**	**1.00**	**1.00**	**1.00**	**1.00**	**1.00**	**1.00**	**1.00**	**1.00**	1.00	1.00
I2: Was there duplicate study selection and data extraction?	0.33	0.71	0.50	0.33	0.61	**0.88**	0.16	0.75	0.20	0.13	0.46	0.42
I3: Was a comprehensive literature search performed?	0.45	0.45	0.45	0.20	0.18	**1.00**	0.29	0.18	0.29	0.29	0.38	0.29
I4: Was the status of publication (i.e. grey literature) used as an inclusion criterion?	0.30	0.19	0.38	0.14	0.48	**0.88**	0.33	0.63	0.54	0.50	0.44	0.43
I5: Was a list of studies (included and excluded) provided?	0.87	0.64	0.75	0.75	0.75	**0.88**	**0.88**	**0.88**	0.63	0.75	0.78	0.75
I6: Were the characteristics of the included studies provided? ^a^												
I7: Was the scientific quality of the included studies assessed and documented? ^a^								0.33				
I8: Was the scientific quality of the included studies used appropriately in formulating conclusions?	**1.00**	**1.00**	−0.07	0.33	**1.00**	−0.07	0.33	−0.07	0.33	−0.11	0.37	0.33
I9: Were the methods used to combine the findings of studies appropriate?	**0.45**	0.19	**0.45**	0.18	0.07	0.33	**0.45**	0.33	0.19	**0.45**	0.31	0.33
I10: Was the likelihood of publication bias assessed?	0.29	0.71	0.53	0.53	0.33	0.73	0.73	0.33	0.33	**1.00**	0.55	0.53
I11: Was the conflict of interest included?	0.24	0.16	0.24	0.20	0.29	0.59	0.35	**0.76**	0.48	0.67	0.40	0.32
Mean	0.55	0.56	0.47	0.41	0.52	**0.69**	0.50	0.53	0.43	0.52	0.52	0.52
Median	0.45	0.64	0.45	0.33	0.48	**0.88**	0.35	0.63	0.33	0.50	0.50	

*I* Item

^a^For item 6 and 7 it was not possible to calculate pairwise inter-rater reliability, except for item 7 pair 3&5, because at least one reviewer of each pair scored “yes” for all 16 reviews (resulting in a constant variable)

Highest values per item are marked in bold, lowest values are marked in italics

While the inter-rater reliability was based on the ratings assigned before the consensus procedure took place, Table 6 presents the frequency with which each reviewer was overruled at item level for AMSTAR. The number of overruled items ranges from 10 to 38. The lowest number of overruled items was found for reviewer 4 (*n* = 10) and reviewer 2 (*n* = 21). This pair of reviewers also had the highest inter-rater reliability.

**Table 6:** Number of overruled assessments for each reviewer at item-level for AMSTAR

	Rev1	Rev2	Rev3	Rev4	Rev5
I1: Was an ‘a priori’ design provided?	0	5	0	0	0
I2: Was there duplicate study selection and data extraction?	5	1	4	0	7
I3: Was a comprehensive literature search performed?	2	1	3	1	2
I4: Was the status of publication (i.e. grey literature) used as an inclusion criterion?	7	3	6	1	5
I5: Was a list of studies (included and excluded) provided?	2	0	2	1	3
I6: Were the characteristics of the included studies provided?	0	0	0	0	3
I7: Was the scientific quality of the included studies assessed and documented?	1	1	3	1	0
I8: Was the scientific quality of the included studies used appropriately in formulating conclusions?	1	1	1	1	4
I9: Were the methods used to combine the findings of studies appropriate?	4	4	7	4	3
I10: Was the likelihood of publication bias assessed?	5	3	11	1	2
I11: Was the conflict of interest included?	7	2	1	0	2
Total	34	21	38	10	29

*I* Item, *Rev* Reviewer

## Discussion

Our study found that inter-rater reliability varies widely depending on the pair of reviewers. Cohen’s kappa ranged from κ = 0.41 to κ = 0.69 (median κ = 0.52) for AMSTAR. This is below the range that was found in the SR dealing with measurement properties of AMSTAR [10]. Inter-rater reliability ranged from κ = 0.50 to κ = 0.87 based on eight studies. Our study results therefore question the validity of previously reported inter-rater reliability measures, assuming a high dependency on the pair of reviewers.

Compared with prior studies, our study showed a lower inter-rater reliability both in general and pairwise. The aforementioned SR reported higher median values of Cohen’s kappa for the 11 individual AMSTAR items, ranging from 0.64 (item 8) to 0.96 (item 11), based on six studies [10]. The median inter-rater reliability in our study ranged from 0.29 (item 3) to 1.00 (item 1). In addition to item 1, only item 5 had a higher inter-rater reliability when compared with the findings from the SR. Items 2, 3, 8 and 11 in our study had a median inter-rater reliability that was even lower than the lowest values in the six studies included in the SR. However, choosing the pair with the highest level of agreement at item level would have yielded higher reliability measures than the SR for all items except item 9 and 11. Comparison with other studies underlines the need for better guidance for rating AMSTAR items. Recently, corresponding proposals have been published based on the assessors’ perspective [21–23].

Although AMSTAR was the main focus of our study, we also included R-AMSTAR for the sake of comparability. The findings were very similar for AMSTAR and R-AMSTAR. Pairs of reviewers with a high inter-rater reliability in AMSTAR typically also had a high inter-rater reliability in R-AMSTAR and vice versa, and the same relationship was observed for all reliability measures. This strengthens the findings of our study as we were able to demonstrate that our finding is not only true for AMSTAR, but also for R-AMSTAR. Having included only one instrument we would not have been able to rule out that our finding is only due to the structure and/or content of AMSTAR. The additional inclusion of R-AMSTAR makes our finding more generalizable. However, one could question whether AMSTAR and R-AMSTAR are too similar in terms of content. Interpretation is further hindered by a lack of papers comparing AMSTAR and R-AMSTAR, as the current evidence is far from clear. In R-AMSTAR’s source publication, a high level of heterogeneity was observed between CRs and nCRs. As a measure of construct validity, the intraclass correlation coefficient (ICC) was ρ = 0.89 (CI: 0.77–0.95) for nCRs and ρ = 0.53 (0.21–0.75) for CRs [9]. Although this was not the main focus of our larger project, our results support this prior finding. Following the same methodology, we obtained a Spearman’s rho of ρ = 0.91 (CI: 0.62–1.00) for nCRs and ρ = −0.12 (−0.89–0.82) for CRs (these results will be presented in another paper). It is obvious that AMSTAR and R-AMSTAR measure the same concept. As already mentioned above, the choice to use R-AMSTAR was made in the context of a larger project. Otherwise, we would have chosen an instrument that measures the same concept (i.e. methodological quality) but is more independent of each other. However, this is not an obvious choice. For instance, the OQAQ was one of multiple tools used to develop AMSTAR. The recently developed ROBIS tool for assessing risk of bias in SRs [24] may be a very interesting comparator. However, the tool has not yet been validated (at the time of this manuscript’s writing), and it also remains unclear whether AMSTAR and ROBIS measure the same concept. While AMSTAR was developed to assess methodological quality, ROBIS focuses on risk of bias in SRs. These are two distinct concepts [25].

Our paper’s conclusion that inter-rater reliability is, to a large extent, dependent on the pair of reviewers would be much stronger still were the same pair of reviewers to show a high inter-rater reliability for several critical appraisal tools while a pair of reviewers with a low inter-rater reliability did not. This would lead to further questions regarding the similarities between the reviewers and a need to take proper account of formal training (e.g. courses at universities or Cochrane workshops), education and other experience. With respect to our results, the pair of reviewers with the highest inter-rater reliability across all reliability measures and both assessment instruments can be described as the pair with the least experience in terms of the number of SRs assessed in the past. However, this does not necessarily reflect the reviewers’ overall experience in critical appraisal, as we did not evaluate such experience in the assessment of primary studies. We based our analysis on experience with the assessment of SRs. However, there may be good arguments for focussing on experience with critical appraisal regardless of the study design. In our study, the years of collaboration between reviewers bore no obvious relation to inter-rater reliability. The pair of reviewers who had worked together for the longest period exhibited only average concordance in their ratings.

Nevertheless, it would be insufficient to focus only on inter-rater reliability, as high inter-rater reliability does not necessarily imply that the ratings are correct. In the absence of an external gold standard to compare our assessments with, we have no choice in our study but to rely on our agreed ratings, assuming these to be “correct”. When the reviewers’ assessments are examined with this in mind, it becomes clear that it is the same raters who had the highest number of correct assessments who also represent the pair of reviewers with the highest inter-rater reliability.

It is worth raising another interesting point regarding the role of the senior author. It was the senior author who introduced the idea for the overall project, and who is leading her working group. Two reviewers involved in this study work in her group. Interestingly, the senior author was overruled less frequently during the consensus procedure. This raises the question whether the professional rank of team members might play a role in the context of critical appraisal. There may be good reasons for this, assuming that a higher position will probably correlate well with experience or expertise in the given field. Accordingly, it cannot be ruled out that the overruling of other team members may reflect a difference in professional rank.

To the best of our knowledge, no study exists with which we could compare our results in the context of critical appraisal scientific literature. However, there are numerous examples that have studied the effect of the pair of reviewers. For example, Hicks et al. compared clinical examination measures for the identification of lumbar segmental instability among three pairs of reviewers and found that the degree of agreement often varied by at least one category [26]. A similar result was found for the etiological classification of ischaemic stroke [27].

In general, there are two possible distinct explanations for reviewer disagreement [28]. The first explanation relates to differences in information, i.e. a relevant piece of information is missed by one or both of the reviewers. The second explanation relates to differences in interpretation, i.e. the reviewers have the same information but reach different interpretations or judgments, as the ratings will probably retain a subjective component. Subjectivity is probably very dependent on the item under study. For example, item 5 *was a list of studies (included and excluded) provided?* is a clear question where the answer can be either yes or no, while item 3 *was a comprehensive literature search performed?* and item 8 *was the scientific quality of the included studies used appropriately in formulating conclusions?* probably will never be a clear yes no, but somewhere between where the reviewer must judge more subjectively. This can also be seen in our results. This is an important point because both situations will lead to a decrease in observer agreement, although only the second relates to the measurement instrument. In our study, we faced both situations: differences in information and differences in interpretation. Although it probably cannot be eliminated altogether, missing information in a report should be kept to a minimum. Therefore, it is usually recommended that assessments are performed by at least two reviewers independently. With respect to the Appraisal of Guidelines for Research and Evaluation II (AGREE II), a critical appraisal tool for the evaluation of clinical guidelines, it is even recommended that assessments are made by four reviewers independently in order to increase reliability [29].

Our study has several limitations. First and probably foremost, although all five reviewers performed their assessments independently, it must be clearly acknowledged that not all of the reviewers were completely independent of one another, as three of them worked at the same centre. One study investigating the reliability of the Risk of Bias (RoB) tool indicated greater agreement between pairs of reviewers from one centre than between four centres [28]. Another study found that RoB assessments were not consistent when the original RoB assessments were compared with external reviewers [30].

Secondly, as our analysis is based only on SRs in occupational health, one could question the generalizability of our results. A prior study indicated a higher level of observer agreement in the case of homogenous studies (i.e. those with the same intervention in similar populations) [31]. Our study had a huge variety in populations and interventions, so it seems unlikely that this has influenced our results. However, our review characteristics closely resemble other very recent studies investigating systematic reviews in different fields [32–34]. It should be noted, though, that the overall AMSTAR score can be treated only as a very rough estimate if used as a measure of methodological review quality, as it has not been formally validated for this purpose [10]. Nevertheless, it is quite useful for descriptive purposes.

Thirdly, the calibration exercise was very short, as only two SRs were included. By selecting one CR and one non-CR, it was possible to point out differences between them. However, both SRs contained meta-analyses. This might have introduced bias, as differences between SRs with quantitative and qualitative syntheses may not have been adequately taken into account, although issues relating to qualitative synthesis were also discussed during the calibration phase. It also became clear during the telephone conferences that, although the protocol for the calibration exercise was available to all reviewers, the reviewers tended towards “rating as usual”. Furthermore, it might also have been interesting to consider additional SR characteristics, such as reporting quality. Unfortunately, no clear guidance exists for calibration exercises in SRs.

Fourthly, our review sample was very small. There is the possibility that our findings are by chance. Our sample size of 16 reviews corresponds to a 25% error margin regarding the raw agreement [35]. Further subgroup analyses were not available and some calculations were not possible due to a too small variability in the data. Additionally, the results might be affected by the type of evidence synthesis (quantitative vs. qualitative), as well as by the publication type (Cochrane reviews vs. non-Cochrane reviews) or the methodological quality of the SRs.

## Conclusions

Better reporting of expertise level is needed in order to improve the interpretability of reliability studies. This also includes information regarding the current and former relationship between the reviewers.

We would welcome further reliability studies. In particular, the distinction between differences in information and differences in interpretation should play a stronger role in future reliability studies. Should the issue of missing information prove problematic, one solution might be to involve more than two reviewers. This choice could also be made according to the expertise level of the reviewers.

## Additional files

Additional file 1: Appendix 1 Search strategy in PubMed. (DOCX 11 kb)

Additional file 2: Appendix 2: overview of included systematic reviews. (DOCX 17 kb)

## Abbreviations

AMSTAR: The Assessment of Multiple Systematic Reviews
CASP: Critical Appraisal Skills Programme
CEBM: Centre for Evidence-based Medicine
COSMIN: COnsensus-based Standards for the selection of health Measurement INstruments
CRs: Cochrane Reviews
nCRs: non-Cochrane reviews
OQAQ: Overview Quality Assessment Questionnaire
PRISMA: Preferred Reporting Items for Systematic Reviews and Meta-Analyses
R-AMSTAR: Revised AMSTAR
SRs: Systematic reviews
