# Supplementary material for: Inter-rater reliability of AMSTAR is dependent on the pair of reviewers
Source: BMC Med Res Methodol. 2017 Jul 11;17:98. doi: 10.1186/s12874-017-0380-y (PMC5504630; doi:10.1186/s12874-017-0380-y)
Supplement: Supplementary file 1 — Appendix 1 Search strategy in PubMed. (DOCX 11 kb) [file 12874_2017_380_MOESM1_ESM.docx]

**Appendix 1**

*Search strategy in PubMed*

(work*[Title] OR occupation*[ Title/Abstract] OR vocational[Title/Abstract] OR employment[Title/Abstract] OR employee[ Title/Abstract]) AND (meta-analysis[Title/Abstract] OR metaanalysis[Title/Abstract] OR "systematic* review*"[Title/Abstract] OR meta-analysis[Publication Type]) AND (random*[Title/Abstract] OR RCT*[Title/Abstract]) AND (intervention* [Title/Abstract] OR program*[Title/Abstract])
