# Supplementary material for: Inter-rater reliability of AMSTAR is dependent on the pair of reviewers
Source: BMC Med Res Methodol. 2017 Jul 11;17:98. doi: 10.1186/s12874-017-0380-y (PMC5504630; doi:10.1186/s12874-017-0380-y)
Supplement: Supplementary file 2 — Appendix 2: overview of included systematic reviews. (DOCX 17 kb) [file 12874_2017_380_MOESM2_ESM.docx]

Appendix 2: overview of included systematic reviews

| **No.** | **Review** | **Topic** | **year** | **CR/ nCR** | **journal** | **RCTs included** | **Meta-analysis** | **AMSTAR score** | **R-AMSTAR score** |
| --- | --- | --- | --- | --- | --- | --- | --- | --- | --- |
| 1 | Sihawong et al. | Exercise therapy for office workers with nonspecific neck pain | 2011 | nCR | J Manipulative Physiol Ther | 9 | no | 7 | 33 |
| 2 | Ojo et al. | Interventions to reduce risky sexual behaviour for preventing HIV infection in workers in occupational settings | 2011 | CR | Cochrane Library | 8 | yes | 10 | 42 |
| 3 | Ng et al. | Seasonal influenza vaccination in healthcare workers | 2011 | nCR | Journal of Hospital Infection | 3 | yes | 7 | 35 |
| 4 | Varatharajan et al. | Work disability prevention for the management of neck pain or upper extremity disorders | 2014 | nCR | J Occup Rehabil | 16 | no | 6 | 34 |
| 5 | Freak-Poli et al. | Workplace pedometer for increasing physical activity | 2013 | CR | Cochrane Library | 4 | yes | 10 | 39 |
| 6 | Cahill und Lancaster | Workplace interventions for smoking cessation | 2014 | CR | Cochrane Library | 57 | yes | 9 | 39 |
| 7 | Parantainen et al. | Blunt versus sharp suture needles for preventing percutaneous exposure incidents in surgical staff | 2011 | CR | Cochrane Library | 10 | yes | 9 | 40 |
| 8 | Elliott et al. | Building capacity and resilience in the dementia care workforce | 2012 | nCR | International Psychogeriatrics | 6 | no | 4 | 32 |
| 9 | Hoving et al. | Non-pharmacological interventions for preventing job loss in workers with inflammatory arthritis | 2014 | CR | Cochrane Library | 3 | no | 9 | 39 |
| 10 | Rongen et al. | Workplace health promotion | 2013 | nCR | Am J Prev Med | 18 | yes | 4 | 29 |
| 11 | Hoe et al. | Ergonomic design and training for preventing work-related musculoskeletal disorders of the upper limb and neck in adults | 2012 | CR | Cochrane Library | 13 | yes | 9 | 39 |
| 12 | Schandel­maier et al. | Return to work coordination programmes for work disability | 2012 | nCR | PLOS ONE | 9 | yes | 8 | 35 |
| 13 | Kinoshita et al. | Supported employment for adults with severe mental illness | 2013 | CR | Cochrane Library | 14 | yes | 10 | 38 |
| 14 | Tan et al. | Preventing the development of depression at work | 2014 | nCR | BMC Medicine | 9 | yes | 5 | 32 |
| 15 | Bauer et al. | Interventions for preventing occupational irritant hand dermatitis | 2010 | CR | Cochrane Library | 4 | no | 9 | 40 |
| 16 | Groeneveld et al. | Lifestyle-focused interventions at the workplace to reduce the risk of cardiovascular disease | 2010 | nCR | Scand J Work Environ Health | 31 | no | 4 | 29 |

CR Cochrane review, nCR non-Cochrane review
